# Effectiveness of Palivizumab in Preventing RSV Hospitalization in High Risk Children: A Real-World Perspective

**DOI:** 10.1155/2014/571609

**Published:** 2014-12-04

**Authors:** Nusrat Homaira, William Rawlinson, Thomas L. Snelling, Adam Jaffe

**Affiliations:** ^1^Disciplines of Paediatrics, School of Women's and Children's Health, UNSW, Sydney Children's Hospital, Level 3, Emergency Wing, Randwick, Sydney, NSW 2031, Australia; ^2^Virology Division, SEALS Microbiology, Prince of Wales Hospital, Randwick, Sydney, NSW 2031, Australia; ^3^School of Medical Sciences, UNSW, Sydney, NSW, Australia; ^4^Biotechnology and Biomolecular Sciences, UNSW, Sydney, NSW, Australia; ^5^Telethon Kids Institute, University of Western Australia, 100 Roberts Road, Subiaco, WA 6009, Australia

## Abstract

Infection with respiratory syncytial virus (RSV) is one of the major causes globally of childhood respiratory morbidity and hospitalization. Palivizumab, a humanized monoclonal antibody, has been recommended for high risk infants to prevent severe RSV-associated respiratory illness. This recommendation is based on evidence of efficacy when used under clinical trial conditions. However the real-world effectiveness of palivizumab outside of clinical trials among different patient populations is not well established. We performed a systematic review focusing on postlicensure observational studies of the protective effect of palivizumab prophylaxis for reducing RSV-associated hospitalizations in infants and children at high risk of severe infection. We searched studies published in English between 1 January 1999 and August 2013 and identified 420 articles, of which 20 met the inclusion criteria. This review supports the recommended use of palivizumab for reducing RSV-associated hospitalization rates in premature infants born at gestational age < 33 weeks and in children with chronic lung and heart diseases. Data are limited to allow commenting on the protective effect of palivizumab among other high risk children, including those with Down syndrome, cystic fibrosis, and haematological malignancy, indicating further research is warranted in these groups.

## 1. Introduction

Globally, respiratory syncytial virus (RSV) is one of the major causes of childhood acute lower respiratory infection (ALRI) associated morbidity, hospitalization, and mortality. In 2005, there were an estimated 3.4 (95% CI 2.8–4.3) million episodes of severe RSV-associated childhood ALRIs necessitating hospital admission [1]. The risk of RSV infection is highest in children born prematurely or those with existing comorbidities including bronchopulmonary dysplasia/chronic lung disease (BPD/CLD), congenital heart disease (CHD), cystic fibrosis (CF), multiple congenital anomalies, and immunodeficiency [2–4].

The reported RSV hospitalization rate in premature infants ranges between 5.2% and 16.8% [5–8]. RSV hospitalization of high risk infants and children is associated with significant health care resource utilization and monetary costs [9, 10]. A high proportion of high risk infants and children hospitalized with RSV infection require admission to an intensive care unit and mechanical ventilation [11]. In the United States it has been estimated that the annual cost of hospitalization for RSV pneumonia in children aged <4 years is approximately $US3 to 4 million [12]. There are additional costs associated with outpatient visits, follow-up, and lost productivity due to parents taking time off work to care for sick children [10].

These factors make prevention of RSV infection a global public health priority, although no active vaccination strategy is yet available. Palivizumab, a humanized monoclonal anti-RSV antibody, was shown to reduce hospitalizations and the clinical severity of RSV infection in high risk infants and children by 55% in a randomised controlled trial [3]. The updated recommendation of the American Academy of Pediatrics (AAP) recommends prophylactic use of palivizumab for children with chronic lung disease of prematurity, congenital heart disease, or other chronic illnesses and for children born at gestational age (GA) less than 29 weeks [13]. The AAP recommends that eligible children should receive no more than five monthly doses of palivizumab [13]. Though guidelines for use of palivizumab for children born with chronic illness are similar across the developed countries, the cut-off gestational age for premature infants varies widely. New Zealand's recommendation is for use in premature infants born at GA ≤ 28 weeks, infants discharged home on oxygen, or those born at GA 29–32 weeks with birth weight of 1000 g or less [14]. The cut-off for GA is <26 weeks in Sweden [15]. Recommendations from Australia and Canada include children born up to 32–35 weeks with two or more risk factors associated with RSV infection [16, 17]. Currently, palivizumab is not routinely recommended for use in children with immune deficiencies, Down syndrome, cystic fibrosis, upper airway obstruction, or other chronic lung diseases.

The cost of a single course of palivizumab is estimated to be as much as $ US4458 per child [18]. Due to its high cost and limited evidence of cost-effectiveness [19], the use of palivizumab is restricted even in well-resourced settings. The cost-effectiveness of palivizumab is largely dependent on its effectiveness across specific subgroups of high risk children.

Several systematic reviews have demonstrated the effectiveness of palivizumab in reducing the subsequent hospitalization rates in high risk children. These reviews have concluded that palivizumab is effective in reducing RSV hospitalization rates by >40% in premature infants and high risk children. However most of these reviews have included efficacy trials conducted under strict controlled environment [20–22]. While randomised trials provide high level evidence of the efficacy of an intervention under idealised conditions, postlicensure observational studies assess the “real-world” value of an intervention under more realistic conditions, where eligibility criteria are less rigidly applied and doses may be delayed or omitted [23]. The objective of this narrative systematic review was to identify which high risk infants and children have been proven to benefit most from palivizumab prophylaxis under real-world practice settings, to identify gaps in existing knowledge, and to provide recommendations for future research.

## 2. Methods

### 2.1. Selection Criteria for the Studies

#### 2.1.1. Study Type

Only full-length peer-reviewed observational studies (cohort, case-control, and cross-sectional studies including survey) published in English were included in the review. As the first randomised trial was published in September 1998 [3], we included studies that were published between January 1999 and August 2013. We excluded conference proceedings, review articles, or editorial reports. We also excluded clinical trial data as we were interested only in the “real-world” effectiveness of palivizumab.

#### 2.1.2. Study Participants

Study participants were high risk infants and children aged <2 years, including premature infants (GA < 37 weeks) and children with any chronic congenital conditions that may put them at risk of severe RSV disease.

#### 2.1.3. Outcome Measure

As the primary interest for this review was the incidence/rate of RSV hospitalization as a measure of prevention of severe RSV disease by immunoprophylaxis with palivizumab, only studies that examined the effect of palivizumab on subsequent RSV hospitalization were included.

### 2.2. Identification of the Studies

We conducted a comprehensive MEDLINE search using the MESH terms “respiratory syncytial virus and primary prevention/immunization,” “respiratory syncytial virus and secondary prevention,” “respiratory syncytial virus and antibodies, monoclonal/or antibodies, monoclonal, humanized/,” “respiratory syncytial virus and hospitalization,” “respiratory syncytial virus and antiviral,” “respiratory syncytial virus and passive immunization,” “respiratory syncytial virus and palivizumab,” and “respiratory syncytial virus and child.” Secondary searches were performed using EMBASE, CINHAL, and Global Health databases using the same keywords. Additional literature was identified by searching the citation list of the identified articles. We also looked for relevant literature using Google Scholar. All the searched results were merged into one single document and all duplicates were removed using Endnote. Once duplicates were removed, we examined the title and the abstract of the literature to exclude articles that did not meet our inclusion criteria. The full length of the relevant articles was retrieved and examined to further determine if they met inclusion criteria.

Each full-length article was reviewed by one researcher (NH) and the summary information extracted using a standardised form following the PRISMA guideline [24]. The data extracted for each article were author, year, and country of publication, time frame of the study, study population, sample size, intervention strategy, dosing regimen, study outcomes, and conclusion. The investigator then shared the abstracted summary form with all other investigators for their review and consensus.

## 3. Results

**Figure 1:**
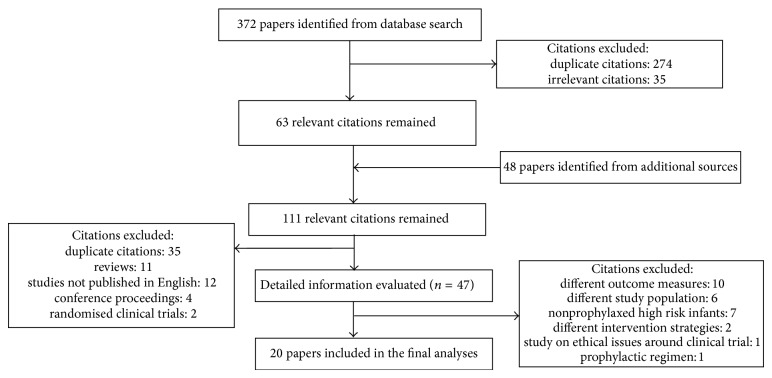
Flow diagram for selection of papers for the review.

**Figure 2:**
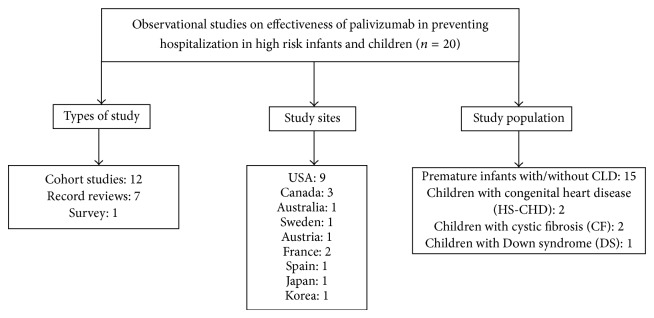
Characteristics of the selected studies on effectiveness of palivizumab in reducing RSV-associated hospitalization in high risk infants and children.

The initial search identified 420 articles (Figure 1). After removing studies that did not meet our inclusion criteria, 20 studies were included: 12 were cohort studies, 7 were record reviews, and one was a questionnaire-based survey. The included studies were conducted in the USA, Canada, Australia, Japan, Korea, and Europe (Figure 2).

### 3.1. Effectiveness of Palivizumab for Preventing RSV-Associated Hospitalization in Premature Infants (Table 1)

Twelve papers [23, 25–35] reported the effectiveness of palivizumab in reducing RSV hospitalization in 89,469 premature infants with or without CLD. Five studies included premature infants born at GA < 36 weeks with or without CLD [23, 26, 28, 34, 35], three studies included only very preterm infants born at GA < 33 weeks [29–31], and four included only premature infants with CLD [23, 25, 30, 34, 36]. Two included studies compared differences in the rate of RSV hospitalization among prophylaxed and nonprophylaxed infants over one RSV season [25, 29], five studies compared rates in prophylaxed and nonprophylaxed groups over noncontemporaneous seasons [23, 26–28, 31], and in two studies rates in prophylaxed infants were compared with rates in nonprophylaxed infants from other published studies [33, 34].

Two of the five studies [26, 28] which included any premature infant born at GA < 36 weeks reported a statistically significant reduction (*P* < 0.05) in RSV hospitalization among prophylaxed compared to nonprophylaxed infants (19–29% rate reduction). Two of the remaining three studies [33, 34] did not have a contemporaneous comparison group or compared the rates to children from other published studies [3, 8]. The remaining study [23] which included late preterm infants (GA 33 to 35 weeks) reported a nonsignificant reduction of 0.6%. Some of the significant risk factors associated with increased risk of RSV hospitalization in premature infants born at GA ≤ 35 weeks included preterm births at GA < 31 weeks, intrauterine growth retardation, single mother family [29], and having siblings at home, more than 5 individuals in the household, and smokers at home [35].

In all of the identified studies which included infants born at GA < 32 weeks, the rate of RSV hospitalization was lower in infants who received palivizumab [27, 29–31, 34]; the reported reduction ranged between 1.2% and 12.4% and in 4 of 5 studies [23, 27, 29, 31] this reduction was statistically significant. Seven of eight studies [23, 25–27, 30, 33, 34, 36] that included premature infants with chronic lung disease reported a statistically significant reduction in children who received palivizumab compared to those who did not; the reduction varied between 0.5 and 29%.

### 3.2. Effectiveness of Palivizumab in Preventing RSV-Associated Hospitalization in Children with Hemodynamically Significant Congenital Heart Disease (HS-CHD) (Table 2)

One study based on hospital record review [37] investigated the effect of palivizumab among 266 children aged <2 years with CHD (Table 2). Rates of RSV hospitalization in prophylaxed children were 19% lower than for nonprophylaxed infants from a different RSV season. The authors concluded the reduction was modest compared to the effect demonstrated in the multinational trial that investigated palivizumab in children with CHD [38] and recommended further assessment of the real-world benefit of palivizumab in this group of children.

### 3.3. Effectiveness of Palivizumab in Preventing RSV-Associated Hospitalization in Children with Cystic Fibrosis (CF) (Table 2)

Two studies included 2966 children with cystic fibrosis [39, 40]. One study compared RSV-associated hospitalization rates between prophylaxed and nonprophylaxed children during the same RSV season, reporting a nonsignificant reduction of 0.2% in the prophylaxed group. A retrospective record review of children with CF listed on a hospital based palivizumab registry reported significantly lower odds of RSV hospitalization in prophylaxed children compared to the reported risk from other published studies.

### 3.4. Effectiveness of Palivizumab in Preventing RSV-Associated Hospitalization in Children with Down Syndrome (Table 2)

One study used data from the Canadian Registry of Palivizumab to compare the rate of RSV hospitalization among 600 children with Down syndrome who received prophylaxis with that of 12,710 children without Down syndrome who received palivizumab [41]. The hospitalization rate in children with Down syndrome (1.53%) was similar to that of other children receiving palivizumab (1.45%).

### 3.5. Association between Dosing of Palivizumab and RSV-Associated Hospitalization (Table 3)

The number of palivizumab doses per child in each RSV season varied between the included studies. Three studies [34, 37, 42, 43] reported an association between the number of doses of palivizumab and the subsequent RSV-associated hospitalization rate in 4,372 children. Palivizumab registry review showed that missed or delayed dosing of palivizumab was associated with statistically significant increase in hospitalization rate in infants who missed or delayed a dose compared to those who were compliant with the scheduled dosing regimen of palivizumab (4.4% versus 2.4%; *P* = 0.02) [34]. A cohort study [43] that included premature infants of GA 29 to 32 weeks also reported a lower RSV hospitalization rate in children prophylaxed according to the standard recommendation of 5 doses, compared with the rate in children who received inadequate prophylaxis, that is, children who did not receive the recommended five full doses (3.3% versus 8.1%, *P* = 0.07). Another cohort study [37] among children with HS-CHD reported a reduction in RSV hospitalizations from 7–9 cases/year when children received palivizumab ad hoc to 2-3 cases/per year when prophylaxis was administered systematically and in accordance with recommendations (*P* = 0.03). One study [44] reported a lower incidence of RSV hospitalization among 17,641 children who received palivizumab at home (0.4%) compared to 1226 children who received it in a clinic (1.2%) (*P* = 0.014).

## 4. Methodological Quality of the Studies

We used the Cochrane GRADE approach to rate the quality of the included studies [45]. The GRADE approach is probably of most relevance for clinical trials as observational studies are inherently prone to bias. However observational studies can nonetheless be upgraded or downgraded based on design, consistency, and precision of results, directness of the evidence, risk of bias, and presence of confounders [45]. Two of the studies included in this review were downgraded to a “very low” quality due to small sample size [25] and lack of a contemporary comparison group [39] limiting the external validity of the study findings. Due to directness of evidence, precision, and comparability of the results, three of the included studies were upgraded to a moderate quality [23, 26, 27] while no other studies required downgrading.

## 5. Discussion

Palivizumab prophylaxis was first shown to reduce RSV-associated hospitalization by 39–78% in premature infants and in children with CLD/BPD in a multinational randomized controlled trial [3]. This landmark trial was subsequently followed by several postlicensure studies in various settings which were included in this review. Our review of these studies found that they also support protective effect of palivizumab against RSV hospitalization among premature infants and in children with CLD, although the size of benefit across the studies was more modest than reported in the clinical trial. Although we did identify studies that reported statistically significant reduction of RSV hospitalization rates among all premature infants with GA < 36 weeks [26, 28], these studies did not stratify the effectiveness in children born at GA 32–35 weeks with additional risk factors. This makes the results inconclusive for premature infants born at GA 32–35 weeks. The only study [23] that stratified by gestational age did not find a significant reduction in hospitalization rate in prophylaxed infants born at GA 33 to 35 weeks without CLD. The review of the studies suggests that groups of children who are likely to benefit most from palivizumab prophylaxis are premature infants born at GA ≤ 32 weeks and children with CLD. Though some international guidelines recommend use of palivizumab in premature infants born at GA < 29 weeks [13–15], highly resourced countries may also consider palivizumab for premature infants born at GA 29–32 weeks as per Canadian and Australian recommendations [16, 17].

One study found a lower rate of RSV hospitalization among children with HS-CHD who received palivizumab [32], a group widely recommended for prophylactic use of palivizumab in international guidelines [13–17]. This reduction, however, was not statistically significant and was much less than the clinical trial that demonstrated a reduction of 45% in children with HS-CHD [38]. The low observed reduction may be attributed to the fact that the study groups were not contemporaneous and RSV transmission may vary across seasons. It may also be that organised and systematic administration of palivizumab is required to achieve a benefit in children with CHD [46]. We identified only one observational study of children with HS-CHD which limits our ability to draw firm conclusion for this group. However as all the guidelines have prioritised children with HS-CHD it may be beneficial to consider these children for five monthly doses of palivizumab during the first RSV season [13].

Three studies [39–41] examined the effect of palivizumab immunoprophylaxis for preventing RSV-associated hospitalization in other groups of high risk children who are not covered by specific recommendations in the AAP guidelines [47], such as children with CF and Down syndrome. These studies did not report a statistically significant reduction of RSV hospitalization rate in these groups of children. In the absence of data from well conducted observational studies demonstrating real-world benefit of palivizumab, international guidelines [13–17] which do not recommend routine use of palivizumab in these groups of children seem appropriate.

We endeavoured to identify all potentially relevant observational studies by using a comprehensive search strategy. As with all systematic search strategies it is possible that we have inadvertently overlooked some relevant studies, for example, unpublished work or studies published in languages other than English. Our review was based on observational studies and inability to completely control for confounding factors is a major limitation of the included studies. In particular “confounding by indication,” in which infants at highest risk of disease are targeted for prophylaxis, has the potential to result in underestimation of the protective effect. In addition many of the included studies compared rates of hospitalization in children receiving prophylaxis with children not receiving prophylaxis from different cohorts and in different RSV seasons and some were conducted over one RSV season. RSV transmission varies over time and geographical location [48, 49] confounding direct interpretation of the results.

We did not include a meta-analysis in our review as the studies included in this review were generally of varying quality, assessed heterogeneous populations, and employed different methodologies limiting quantitative synthesis. The review of the included studies prevents us from drawing firm conclusions except for among preterm infants (GA < 33 weeks) and those with CLD and HS-CHD. Further studies of palivizumab in children with Down syndrome, CF, or hematologic malignancies and other high risk groups may inform the current guidelines for immunoprophylaxis. Also studies documenting the association between number of palivizumab injections and RSV infection may better define the optimal dosing of the immunoprophylaxis with respect to burden and cost.

## 6. Authors' Conclusion

RSV continues to be one of the major causes of childhood hospitalization worldwide. This review supports use of palivizumab for premature infants born at gestational age < 33 weeks and in selected subgroups of children at high risk of developing severe RSV disease, in particular children with CLD or HS-CHD. Recommendations for targeted immunoprophylaxis for specific groups at high risk for severe RSV disease need to be based on better effectiveness data, along with country-specific data on burden of RSV disease, prevalence of risk factors, and the availability of funds for preventive interventions.

## Figures and Tables

**Table 1:** Studies on effectiveness of palivizumab in reducing RSV-associated hospitalization in premature infants with or without congenital lung disease (CLD).

Author, year, and location	Study design	Timeline	Study population	Intervention strategy	Sample size	RSV hospitalization rates	Conclusion	Quality of study
(1) Sorrentino and Powers, 2000, USA [33]	Retrospective chart review	1998-1999	Premature infants with GA ≤ 35 weeks and age <2 years with CLD	Palivizumab only	1839	Infants without CLD = 2.1%, with CLD = 4.0%, and with CF = 0%	Hospitalization rates in prophylaxed children were similar to rates in prophylaxed children from IMpact-RSV trial [3]	Low

(2) Singleton et al., 2003, Alaska [26]	Cohort study	1993–2001	Premature infants GA < 36 weeks	Prepalivizumab era (1993–1996) versus postpalivizumab era (1998–2001)	Prepalivizumab era: 992 Postpalivizumab era: 1087	Prepalivizumab era = 439/1000 births Palivizumab era = 150/1000 births (*P* < 0.01)	The rate decreased by 3-fold with palivizumab	Moderate

(3) Pedraz et al., 2003, Spain [27]	Cohort study	1998–2000 and 2000–2002	Premature infants with GA ≤ 32 without CLD and children aged <2 years with CLD	Prepalivizumab era (1998–2000) versus postpalivizumab era (2000–2002)	Nonprophylaxed group: 1583 Prophylaxed group: 1919	Infants ≤ 28 week GA: nonprophylaxed = 13%, prophylaxed = 5.4% (*P* < 0.0001) Infants 29–32 week GA: nonprophylaxed = 9.9%, prophylaxed = 2.5% (*P* < 0.0000) Infants with CLD: nonprophylaxed = 19.7%, prophylaxed = 5.5% (*P* < 0.007)	Effective for children with GA ≤ 32 weeks and for children with CLD	Moderate

(4) Lacaze-Masmonteil et al., 2004, France [29]	Cohort study	2000-2001	Premature infants with <33 week GA	No palivizumab versus palivizumab	Nonprophylaxed group: 2370 Prophylaxed group: 376	Nonprophylaxed = 20.1% Prophylaxed = 8.4% (*P* < 0.001)	Protective benefits for infants with GA < 33 weeks	Low

(5) Parnes et al., 2003, USA [34]	Record review	2000-2001	All children eligible for palivizumab according to AAP guidelines [40]	Received at least one dose of palivizumab	2116 GA < 32 weeks: 986 (46.6%) GA 32– 35 weeks: 957 (45.2%) GA >35 weeks: 172 (8.1%)	All infants = 2.9% Infants with CLD = 5.8% Infants < 32 weeks of GA = 4.5% Infants > 35 weeks of GA = 0.6%	RSV hospitalization rates lower compared to rate of 13% in nonprophylaxed children from Spain [8]	Low

(6) Henckel et al., 2004, Sweden [30]	Cohort study	1992–2002	Preterm infants with CLD and age < 2 years and extreme preterm infants	No palivizumab versus Palivizumab	Nonprophylaxed group: 61,990 Prophylaxed group: 235	Infants with CLD: nonprophylaxed = 6.8%, prophylaxed = 7.3% (*P* = 0.91) Infants with GA < 33 weeks: nonprophylaxed = 3.9%, prophylaxed = 2.7% (*P* = 0.61)	Use of palivizumab may be restricted to very preterm infants (GA < 26 weeks) suffering from severe CLD	Low

(7) Singleton et al., 2006, Alaska [28]	Cohort study	1994–1997 and 2001–2004	Premature infants GA < 36 weeks	Prepalivizumab era (1994–1997) versus postpalivizumab era (2001–2004)	2555 children	Prepalivizumab era = 317/1000 births/year Palivizumab era = 123/1000 births/year (*P* < 0.001)	Palivizumab reduced hospitalization rate in premature infants	Low

(8) Mitchell et al., 2006, Canada [23]	Cohort study	1995–2002	All premature infants	Prepalivizumab era (1995–1998) versus postpalivizumab era (1999–2002) (in Calgary palivizumab was introduced in 1999 whereas in Edmonton palivizumab was not administered)	Calgary: 2,876 GA 33–35 weeks: prepalivizumab era: 907, postpalivizumab era: 842 GA < 33 weeks or with CLD: prepalivizumab era: 411, postpalivizumab era: 496 Edmonton: 2,467 GA 33–35 weeks: prepalivizumab era: 787, postpalivizumab era: 834 GA < 33 weeks or with CLD: prepalivizumab era: 401, postpalivizumab era: 425	Edmonton = 7.1% Calgary = 2.9% (*P* = 0.004) Calgary Infants with GA < 33 weeks or GA 33–35 weeks with CLD: prepalivizumab era = 7.3%, postpalivizumab era 3.0% (*P* = 0.003) Infants with GA 33–35 weeks and born within 6 months of RSV season or at the start of RSV season: prepalivizumab era = 3.3%, postpalivizumab era = 2.7% (*P* = 0.38) Edmonton Infants with GA < 33 weeks or GA 33–35 weeks with CLD: prepalivizumab era = 5.0%, postpalivizumab era = 7.0% (*P* = 0.212) Infants with GA 33–35 weeks: prepalivizumab era = 4.1%, postpalivizumab era = 2.1% (*P* = 0.02)	Effective for infants with GA < 33 weeks with other comorbidities Could be upgraded to moderate	Moderate

(9) Kusuda et al., 2006, Japan [25]	Nonrandomised questionnaire survey	2002-2003	All premature infants born at GA ≤ 35 weeks and infants with CLD	No palivizumab versus palivizumab	Nonprophylaxed group: 41 Prophylaxed group: 35	Nonprophylaxed group = 1.3% Prophylaxed group = 1.4%	Small sample size to determine conclusive result	Very low

(10) Grimaldi et al., 2007, France [31]	Cohort study	1999–2004	Premature infants with GA ≤ 30 weeks without BPD	Prepalivizumab era (1999–2002) versus postpalivizumab era (2002–2004)	Prepalivizumab era: 118 Postpalivizumab era: 88	Prepalivizumab era = 13.5% Postpalivizumab era = 1.1% (*P* < 0.0001)	Palivizumab reduced hospitalization rate	Low

(11) Chang et al., 2010, Korea [36]	Hospital record review	2004–2009	Children were born at ≤35 weeks of GA, were <2 years of age, and had received medical therapy for CLD	No palivizumab versus palivizumab	Nonprophylaxed group: 53 Prophylaxed group: 75	Nonprophylaxed group = 22.6% Prophylaxed group = 4.0% (*P* < 0.001)	Palivizumab reduced RSV hospitalization in children with CLD	Low

(12) Paes et al., 2012, Canada [35]	Cohort study	2006–2011	Group 1: premature infants GA ≤ 32 weeks without preexisting medical disorders Group 2: premature infants GA 33–35 completed weeks	Comparison between prophylaxed Group 1 and prophylaxed Group 2	Group 1: 5,183 Group 2: 1,471	Group 1 = 1.5% Group 2 = 1.4% (*P* = 0.3)	Palivizumab prophylaxis beneficial for infants with 33–35 week GA but should be country-specific	Low

**Table 2:** Studies on effectiveness of palivizumab in reducing RSV-associated hospitalization in children with congenital anomalies.

Author, year, and location	Study design	Timeline	Study population	Intervention strategy	Sample size	RSV hospitalization rate	Conclusion	Quality of study
Studies on effectiveness of palivizumab in children with hemodynamically significant congenital heart disease (HS-CHD)								
(13) Chang and Chen, 2010, USA [32]	Hospital record review	2000–2006	Children aged <2 years with HS-CHD	Prepalivizumab era (2000–2003) versus postpalivizumab era (2004–2006)	266 children with HS-CHD	19% reduction, HS-CHD patients comprised of 0.56% of all RSV hospitalization in prepalivizumab era and 0.46% in palivizumab era	Requires further investigation of cost-effectiveness of palivizumab in children with HS-CHD	Low

Studies on effectiveness of palivizumab in children with cystic fibrosis (CF)								
(14) Winterstein et. al., 2013, USA [40]	Cohort study	1999–2006	Children < 2 years with CF	No palivizumab versus palivizumab	Nonprophylaxed group: 575 Prophylaxed group: 2300	Nonprophylaxed group = 4.1 (2.8–6.0)/1000 season months Prophylaxed group = 2.4 (0.8–6.6)/1000 season months	Potential benefit inconclusive	Low

(15) Speer et al., 2008, USA [39]	Palivizumab registry review	2000–2004	Infants and young children with CF	No palivizumab in historical cohort versus palivizumab in study population	91	Significant reduction in hospitalization (compared to Abman et al. [50]: odds ratio = 33.07, 95% CI = 1.844–593.2, *P* < 0.0004; Armstrong et al. [51]: odds ratio, =18.67, 95% CI = 1.048–332.6, *P* < 0.0042)	Further investigation for usefulness of RSV in children with CF	Very low

Studies on effectiveness of palivizumab in children with Down syndrome (DS)								
(16) Paes et al., 2013, Canada [41]	Record review of the palivizumab registry	2006–2012	High risk infants receiving at least one dose of palivizumab	Effect of palivizumab in children with DS compared to all other children	13,310 children of which 600 were with DS	Hospitalization rate among prophylaxed DS children was 1.53% and similar to children with other standard indications (1.45%)	DS children aged <2 years should be considered candidates for palivizumab	Low

**Table 3:** Studies on association between palivizumab dosing and RSV-associated hospitalization.

Author, year, and location	Study design	Timeline	Study population	Intervention strategy	Sample size	Dose of palivizumab	RSV hospitalization rate	Conclusion	Quality of study
(17) Forgel et al., 2008, USA [44]	Palivizumab outcome registry review	2000–2004	High risk infants and young children eligible for palivizumab	Association between rates of RSV hospitalization and site of palivizumab administration	17,641 in clinic setting and 1226 in home setting	88% in home setting and 81% in clinic setting received the appropriate number of dosing	Received palivizumab at home: 0.4% (5/1226) Received palivizumab in clinic: 1.2% (207/17,641) (*P* = 0.0139)	Home administration of palivizumab may be preferred for high risk infants at risk of RSV hospitalization	Low

(18) Palivizumab Outcomes Registry Study Group, 2003, USA [34]	Record review	2000-2001	All children eligible for palivizumab according to AAP guidelines [40]	Received at least one dose of palivizumab	2,049	1,638 of 2,049 (80%) children were compliant with the scheduled dosing of palivizumab 472 (23%) of 2,049 infants missed or had a delay in receiving an injection	RSV hospitalization slightly higher in noncompliant infants (3.4% versus 2.8%, *P* = 0.48) Hospitalization rate significantly higher in infants who missed or delayed an injection (4.4% versus 2.4%) (*P* = 0.020)	Missed or delayed palivizumab injections may increase the incidence of hospitalization	Low

(19) Resch et al., 2006, Austria [43]	Cohort study	2001–2003	Premature infants of GA 29–32 weeks with and without BPD	Comparison between infants receiving adequate dosing of palivizumab and those receiving at least 1 dose of palivizumab	238 children received palivizumab	Mean number of injections/child 2.5 ± 1.6	Adequate prophylaxis: 3.3% Inadequate prophylaxis: 8.1% (*P* = 0.07)	Missed or delayed palivizumab injections may increase the incidence of hospitalization	Low

(20) Chadha et al., 2012, USA [42]	Cohort study	2005–2009	Premature infants < 32 week GA	Relation between different dosing rate of palivizumab and hospital admission	1965 infants	0 doses: <29 week GA = 42%, 29–31 week GA = 39.8% At least 1 dose: <29 week GA = 58%, 29–31 week GA = 60.2% Full dose: <29 week GA = 14.8%, 29–31 week GA = 17.6%	Weak positive correlation between palivizumab dosing and hospital admissions *P* = 0.057 Spearman rho = 0.012	Overall reduced dosing of palivizumab and seasonal variation in severity of RSV disease may have affected the results	Low

(21) Alexander et al., 2012, Australia [37]	Cohort study	2005–2009	Infants with HS-CHD	Patients who received palivizumab on ad hoc basis (2005–2007) versus patients who received it systematically (2008-2009)	120 (3 in 2005–2007 and 117 between 2008 and 2009)	2005–2007: mean 1-2/child 2008-2009: mean 4/child	2005–2007: 7–9 patients/year 2008–2009: 2-3 patients/year (*P* = 0.03)	Systematic administration of palivizumab reduced hospitalization rates	Low
